# Our experience with open dismembered pyeloplasty for uretero-pelvic junction obstruction

**DOI:** 10.12669/pjms.301.3201

**Published:** 2014

**Authors:** Shahnawaz, Ali Shahzad, Iqbal Shahzad, Muhammad Umar Baloch

**Affiliations:** 1 Dr. Shahnawaz, MS,; 2 Dr. Shahzad Ali, FCPS,; 3 Dr. Iqbal Shahzad, FCPS,; 4 Dr. Muhammad Umar Baloch, MS,

**Keywords:** Uretero-pelvic Junction Obstruction, Open Pyeloplasty, IVU, DTPA

## Abstract

***Objective:*** **To report** our experience with open dismembered pyeloplasty for uretero-pelvic junction obstruction.

***Methods: ***Retrospective study was conducted in the Department of Urology, Jinnah Postgraduate Medical Centre, Karachi for a period of five and half years from May, 2006 to December, 2011. All patients with uretero-pelvic junction obstruction were entered into a database to record patients clinical features, diagnostic tools, operative and post-operative details and follow-up. Over a five-years period, 13 procedures were performed. After clinical evaluation all patient had extensive haematological and radiological workup for diagnosis of uretero-pelvic junction obstruction. All were subjected to open pyeloplasties, out of these 13 patients; one had an aberrant lower pole vessel compressing uretero-pelvic-junction. All procedures were stented. Repair was done with 3/0 vicryl sutures all patients were catheterized and wound drained.

***Results: ***Mean operating time was 60 – 100 minutes with about 100cc blood loss requiring no transfusion. The mean follow up was one year. One patient developed post-operative haematuria and was managed conservatively. Two patients developed fever secondary to urinary tract infection despite adequate treatment of urinary tract infection according to culture and sensitivity pre-operatively. One patient developed surgical emphysema detected post-operatively, which required tube thoracostomy. Neither patient developed recurrent symptoms nor had any evidence of obstruction on the renogram on follow-up. Objectively all patients were followed up by intravenous urogram, stress renogram, Urine C/S. Subjective and objective follow-up revealed success in 100% of patients whereas success is defined as no or minimal holder on DTPA renogram, improving renal function and decreasing dilatation on successive intravenous urogram. All patients had a mean post-operative hospital stay of 02 – 04 days Folley catheter was removed after 10-days, double-j- stents were removed after two to three weeks.

***Conclusion: ***Our success rate following open pyeloplasty with limited follow-up was 100%. It is comparable with International data. Recent international trend is toward Uretro-pelvic Junction Obstruction (UPJO) repair with laparoscopic approach, they are claiming success rate of 95%.

## INTRODUCTION

The surgical management of uretero-pelvic junction obstruction has undergone revolutionary changes over the past few years. Traditionally, open retroperitoneal dismembered reduction pyeloplasty has been considered as the treatment of choice for uretero-pelvic junction obstruction with high success rates of over 95%.^1^ However the procedure requires a longer flank incision and an associated longer recovery period.^1^ Endopyelotomy became popular in the late 1980's and early 1990's as a minimally invasive technique with lower complication rates, relatively shorter operating times and quick recovery.^1^

The success rates quoted in literature range between 63% to 93%^2^^-^^4^ in well-selected patients. Excellent success rates have been found in patients with a smaller pelvis and in whom no crossing vessels were present. By the end of the last decade, laparoscopic pyeloplasty made its place and is now becoming popular day by day. Success rates are repoted in between 87–100%.^2^^,^^3^^,^^5^^-^^9^ The procedure allows the identification of crossing vessels, excision of the pathological uretero-pelvic junction segment ± a reduction pyeloplasty and a water tight anastomosis over a stent. In addition, there is less pain with short hospital stay and a quick recovery period.^1^ However, the procedure requires skill, a long learning curve and involves longer operating times as compared to open and minimally invasive techniques. Laparoscopic pyeloplasty can be performed via a retroperitoneal or transperitoneal approach. Equivalent success rates have been quoted in the literature.^5^^,^^8^^-^^11^ We analysed the data of all open pyeloplasty were performed since 2006 to report our experience and the successful outcome we achieved.

## METHODS

All patients presenting to our institution with uretero-pelvic junction obstruction after May, 2006 are entered into a database. Patients details, operative information and post-operative follow up were recorded.

Over this five-year period, a total of 13 procedures were performed for uretero-pelvic junction obstruction. All procedures were open retroperitoneal dismembered reduction pyeloplasties performed by single surgeon. One (01) patient had a double-j-stent inserted initially as temporary measure pre-operatively.

There were 08 males and 05 females (male to female ratio 8:5) included in the study. The left kidney was most commonly affected. Loin pain was the predominant presenting symptom in 76.9% with 15.38% presenting with recurrent urinary tract infections and 7.69% presented with haematuria. Three patients (23.07%) were also noted to have concurrent renal calculi pre-operatively. The mean age at the time of operation was 22 (16–35) years.

***Open dismembered reduction pyeloplasty technique:***

***Preparation and positioning: ***A decision about the need for pre-operative retrograde pyelography is always made on individual basis. A folley catheter is placed in the bladder and the patient positioned with the affected side up to make a lumber subcostal / 12th rib approach whatever required.

***Procedure: ***The retroperitoneal space was initially developed by blunt dissection to push the peritoneum away. The ureter was identified and traced upto the uretero-pelvic junction.

Kidney and proximal ureter were mobilized taking care not to damage blood supply of proximal ureter. Once uretero-pelvic junction was exposed, fine stay sutures were placed at the anterior portion of proximal ureter so that it might not twist and at the upper and lower ends of pelvis. The proximal ureter was then transected above the marking suture. The renal pelvis was transected similarly in a diamond shape manner. Now the obstructed UPJ was separated out. Repair was carried out using 3/0 vicryl interrupted sutures. We did reduction of the renal pelvis in all cases. Aberrant lower pole vessel found in one case as the cause of external compression, was not disturbed. Rather UPJ repair was done posterior to it. Anastomosis was always stented with double-j-stent and perinephric space was also always drained. Folley was also inserted.

***Postoperative care: ***The drain was removed on the 3rd postoperatve day and folley catheter is usually removed on the tenth postoperative day. The stent is removed in two to three weeks’ time. An F-15 diuretic MAG-3 renogram is performed at 3 and 12 months, and annually thereafter. When this is equivocal, an IVU is performed and urine culture sensitivity is done.

Success is defined as no or minimal hold-up on DTPA renogram, improving renal function and decreasing dilatation on successive intravenous urogram.

## RESULTS

All the 13 patients underwent open dismembered reduction pyeloplasty. Twelve (92.30%) patients were treated primarily with pyeloplasty and one patient treated with double-j-stent upon her choice after adequate counselling but eventually procedure of pyeloplasty was done.

There were 08 males and 05 females (male to female ration 8:5) included in the study. The left kidney was most commonly affected. Loin pain was the predominant presenting symptom in 76.9%, the recurrent urinary tract infections in 15.38% and haematuria in 7.69%. Three patients (23.07%) were also noted to have concurrent secondary renal calculi pre-operatively. The mean age at the time of operation was 22 (16–35) years +6.

The mean operative time was 60–100 minutes. Two (15.38%) patients developed fever and urinary tract infection although had an adequate per-operative antibiotic coverage according to culture and sensitivity. One (7.69%) patient developed haematuria which was conservatively managed. One (7.69%) patient developed surgical emphysema which was detected post-operatively and a tube thoracostomy was made. None of our patients developed recurrence even after two years of follow-up. The Radio-nuclide stress renogram, urine C/S and intravenous urogram / ultrasonography and renal function profile were used to record improvement in all of our patients.

This is in accordance to different institutions database, the success rate for open pyeloplasty at their institution is 95-97% with a mean follow-up of 48 months and the mean post-operative stay is (2 – 4) days.

None of the patients in our series developed recurrence or failure. We believe that this could be due to small size of the study and probably a longer followup might further enlighten our higher success rate.

**Table-I:** Open dismembered reduction pyeloplasty

	*No. of patients (n=13)*
Male	08
Female	05
Age years (range)	22 (16–35)

*Side of kidney:*	
Left	08
Right	05


**Table-II:** Peri-operative details for open retroperitoneal dismembered reduction pyeloplasty

Mean operative time / mins (range)	80 (80 – 100)
Mean post-op stay / days (range)	3.0 (2 – 4)
Pre-op function/% (range)	33 (20 – 54%)
Post-op function/% (range)	35 (17 – 56%)
Mean follow-up / months (range)	24
Success rate/n (%)	13 (100%)

## DISCUSSION

No doubt, >90% success rate have been observed with gold standard surgical approaches whether it be open or laparoscopic pyeloplasty for uretero-pelvic junction obstruction.^12^ Traditional transanastomotic stenting also acting as a nephrostomy tube drainage of the kidney has been replaced by double–j-stenting of the uretero pelvic junction.^13^ Although non stented pyeloplasty has been used in paediatric patients with similar results to stented pyeloplasty.^14^ In third world countries like Pakistan laparoscopicaly is not yet performed frequently. Therefore we performed open dismembered reduction pyeloplasty in our tertiary care referral centre.

Reviewing our more than five years data of open dismembered pyeloplasties, a success rate of 100% is seen at a mean follow-up of 12 months. Whereas success is defined as no or minimal holdup on DTPA renogram, improving renal function and decreasing dilatation on successive intravenous pyelograms. It is equivalent to that seen with other international open pyeloplasty series.^1^ Our mean follow-up is short; however other series report that failures following open retroperitoneal pyeloplasty tend to occur within the first post-operative year.^6^

**Fig.1:**
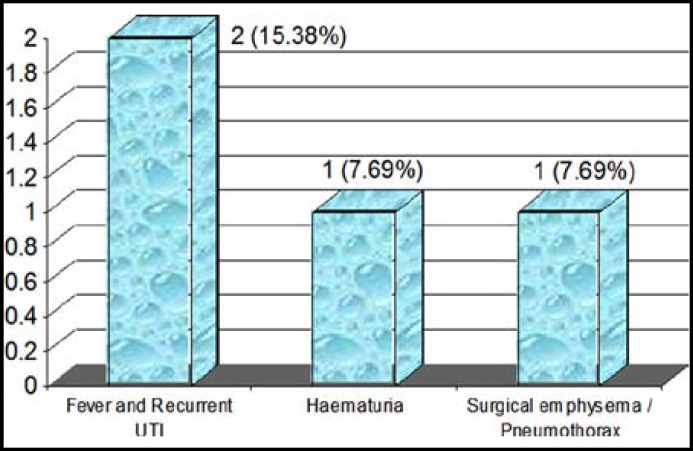
Post-operative complications.

The mean operative time for our series patients was 80 minutes; however it varied between 60-130 minutes with majority of cases finishing by 80 minutes. Mean operating time in different institutions varies from 60 – 100 minutes. Our institution is dedicated to teaching trainees and this may further add to longer operative time in few cases. In addition, all of our patients required a reduction pyeloplasty, thus increasing operative time. According to national and international literature, almost equivalent success rates (95 – 100%) are reported for this procedure.^5^^,^^8^^-^^11^ Only one patient had an aberrant crossing vessel to lower pole compressing uretero-pelvic-junction.

The main advantage with the open retroperitoneal approach is the reduced risk of bowel injury and the better familiarity of retro peritoneum to urologists. Crossing vessels may be easily visualised, the anastomosis may be transposed anterior to the crossing vessels to minimise tension on the anastomosis. A reduction pyeloplasty is also more technically feasible where necessary. Often trainee urologists are first introduced to other retroperitoneal procedures like open pyelolithotomy and retroperitoneal nephrectomies and then exposed to open retroperitoneal pyeloplasties. Experience reduces complications and the time duration of the procedure and success rate also increases.

The disadvantages include large access wound, weak postoperative muscle strength mimicking hernia, difficulties with orientation and limited space which can be multiplied by excessive retroperitoneal fats. Suturing can be difficult, especially with inadequate lateral positioning of the patient.^1^ Several authors including us have reproduced the high success rates achieved with open surgery with low morbidity and early discharge from the hospital.^15^^,^^16^

Advances in equipment and technique have resulted in technique shift in the favour of laparoscopic pyeloplasty. The main argument against laparoscopic reconstructive procedures is because laparoscopic suturing is challenging, time consuming, and is associated with prolonged learning curve.^16^

 Regarding laparoscopic procedures we are still in learning phase, hopefully in near future we will be able to perform laparoscopic pyeloplasty and compare our results with open technique. Our study has some limitation like shorter follow-up, small sample size, overage group and non-laparoscopic pyeloplasty surgical technique.

## CONCLUSION

Overall our success rate following open retroperitoneal dismembered reduction pyeloplasty is 100%, which is equivalent to that seen from other Centres with the additional benefits of reduced hospital stay. Regarding laparoscopy we are still in learning phase, hopefully in near future we will be able to perform both open and laparoscopic pyeloplasty at our Centre and would be able to compare our results accordingly.

## Authors Contribution:

Shahnawaz conceived, designed and collected data and performed surgery,

SA did statistical analysis, manuscript writing and surgery,

IS did editing of manuscript,

MUB was involved in review and final approval of manuscript.

## References

[1] Bauer JJ, Bishoff JT, Moore RG, Chen RN, Iverson AJ, Kavoussi LR (1999). Laparoscopic versus open pyeloplasty: assessment of objective and subjective outcome. J Urol.

[2] Parkin J, Evans S, Kumar PVS, Timoney AG, Keeley Jr FX (2003). Endoluminal ultrasound before retrograde endopyelotomy: Can the results match laparoscopic pyeloplasty?. BJU Int.

[3] Pardalidis NP, Papatsoris AG, Kosmaoglou EV (2002). Endoscopic and laparoscopic treatment of uretero-pelvic junction obstruction. J Urol.

[4] Albani JM, Yost AJ, Streem SB (2004). Uretero-pelvic junction obstruction: determining durability of endourological intervention. J Urol.

[5] Eden CG, Cahill D, Allen JD (2001). Laparoscopic dismembered pyeloplasty: 50 consecutive cases. BJU Int.

[6] Jarrett TW, Chan DY, Charambura TC, Fugita D, Kavoussi LR (2002). Laparoscopic pyeloplasty. The first 100 cases. J Urol.

[7] Turk IA, Davis JW, Winklemann B, Deger S, Richter R, Fabrizio MD (2002). Laparoscopic dismembered pyeloplasty. The method of choice in the presence of an enlarged renal pelvis and crossing vessels. Eur Urol.

[8] Soulie M, Salomon L, Patard JJ, Mouly P, Manuta A, Antiphen P (2001). Extraperitoneal laparoscopic pyeloplasty: A multicentre study of 55 procedures. J Urol.

[9] Hemal AK, Goel R, Goel A (2003). Cost effective laparoscopic pyeloplasty: Single centre experience. Int J Urol.

[10] Deger S, Roigas J, Wille A, Giessing M, Schonberger B, Turk IA (2003). Laparoscopic dismembered pyeloplasty with Anderson-Haynes technique. Urologe A.

[11] Gnanapragasam VJ, Armitage TG (2001). Laparoscopic pyeloplasty, initial experience in the management of UPJO. Ann R Coll Surg Eng.

[12] Ozdemir T, Arikan A (2010). One day hospitalization after open, double-J-stented pyeloplasty. World J Paediatr.

[13] Chandrasekharam VV ( 2005). Is reterograde stenting more reliable than antegrade stenting for pyeloplasty in infants and children?. Urology.

[14] Smith KE, Holmes N, Leib JI, Mandell J, Baskin LS, Kogan BA (2002). Stented versus non-stented paediatric pyeloplasty: A modern series and review of the literature. J Urol.

[15] Yanke BV, Lallas CD, Pagnam C (2008). The minimally invasive treatment of uretero-pelvic-junction obstruction: a review of our experience during the last decade. J Urol.

[16] Piaggio LA, Guimond JF, Noh PH (2007). Transperitoneal laparoscopic pyeloplasty for primary repair of uretero-pelvic-junction obstruction in infants and children: comparison with open surgery. J Urol.

